# Letter from the Editor in Chief

**DOI:** 10.19102/icrm.2022.130102

**Published:** 2022-01-15

**Authors:** Moussa Mansour


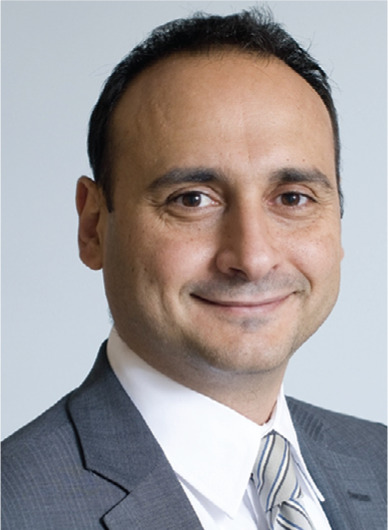


Dear Readers,

The 27^th^ Atrial Fibrillation (AF) Symposium was held this month in New York City. Despite challenging circumstances imposed by the coronavirus disease 2019 pandemic, there were a significant number of in-person attendees and a large online audience. This year, like in previous years, the newest and most recent developments in the field of AF were presented by scientists from the United States and abroad.

One area of the AF Symposium that has been gathering significant attention over the past few years is the late-breaking clinical trials session (LBCT). This year, 7 landmark studies were presented during this session, and I would like to highlight 3 of them here.

The first study was presented by Dr. Vivek Reddy and was titled “Clinical Outcomes of Lattice-tip Focal Ablation for Atrial Fibrillation: Toggling Between Pulsed Field and Radiofrequency Energy.” It reported the long-term clinical outcomes of safety and efficacy of ablation using a novel lattice-tip catheter. One hundred seventy-eight patients with paroxysmal (44%) or persistent (56%) AF were enrolled and underwent ablation using the 9-mm spherical multielectrode lattice-tip study catheter. Pulsed-field ablation was used when ablating the posterior left atrial wall, and radiofrequency ablation was used elsewhere. In addition to pulmonary vein (PV) isolation (PVI), mitral isthmus, cavo-tricuspid isthmus, and left atrial roof ablation were performed in 44%, 70%, and 73% of cases, respectively. The clinical outcomes were excellent from many perspectives, including the fluoroscopy time, which was only 4.3 ± 3.1 minutes. A remapping study performed at 96 ± 43 days on a subgroup of patients confirmed a 97% rate of durable PVI when an optimal electroporation waveform was used. The same waveform resulted in an amazing 84.5% ± 5.1% rate of freedom from atrial arrhythmias at 308 days of follow-up. Most importantly, just 1 primary adverse event occurred during the trial (inflammatory pericardial effusion not requiring intervention); there were no cases of esophageal fistula, stroke/transient ischemic attack, phrenic injury, or PV stenosis.

The second LBCT I wish to cover is the First In-human Experience and Acute Procedural Outcomes using a Novel Pulsed-field Ablation System (PULSED AF) pilot trial, which was presented during the symposium by Dr. Atul Verma. This study was a non-randomized prospective multicenter clinical trial that enrolled 35 patients with paroxysmal or persistent AF who underwent PVI using the novel PulseSelect multielectrode circular pulsed-field ablation system (Medtronic, Minneapolis, MN, USA). The results of this study were impressive, demonstrating acute PVI in all patients, and no serious adverse events were recorded during the 30-day follow-up period, including phrenic nerve injury, esophageal injury, stroke, and death. The study was simultaneously published in *Circulation: Arrhythmia and Electrophysiology.*^1^

The third LBCT of interest is the prospective, non-randomized, multicenter 12M Safety/Effectiveness of Very-high-power Short-duration Pulmonary Vein Isolation with a Contact Force–sensing, Temperature-controlled Radiofrequency Catheter (Q-FFICIENCY) trial, which was presented by Dr. Jose Osorio. This study reported experience with a contact force–sensing catheter optimized for temperature-controlled radiofrequency ablation using 6 thermocouples. The system allows power modulation to maintain a target temperature and thus has the ability to deliver very-high-power, short-duration (90-W, 4-second) ablation lesions. One hundred sixty-six patients underwent ablation and were followed for 12 months. During the follow-up period, 77% of patients remained free from atrial arrhythmias, and no case of device- or procedure-related death, atrioesophageal fistula, stroke, transient ischemic attack, or severe PV stenosis was observed.

In addition to the 3 abovementioned LBCTs, numerous other studies were presented, including other first-in-man procedures. Moreover, 14 recorded and live case transmissions were shown, which provided a comprehensive view of the recent trends in the field of AF ablation and left atrial appendage closure.

I hope that you enjoy reading this issue of *The Journal of Innovations in Cardiac Rhythm Management*, and best personal regards.

Sincerely,


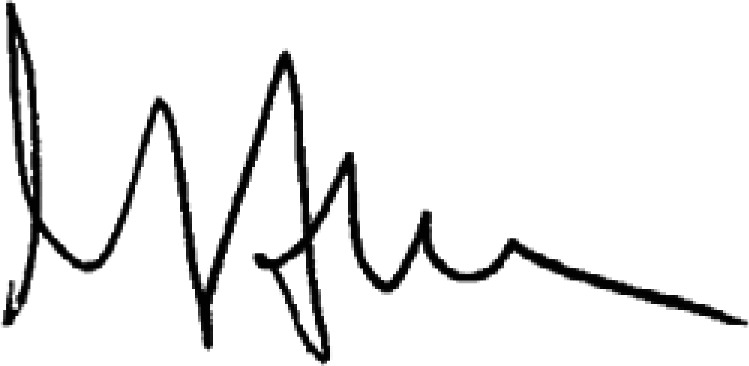


Moussa Mansour, md, fhrs, facc

Editor in Chief

*The Journal of Innovations in Cardiac Rhythm Management*

MMansour@InnovationsInCRM.com

Director, Atrial Fibrillation Program

Jeremy Ruskin and Dan Starks Endowed Chair in Cardiology

Massachusetts General Hospital

Boston, MA 02114
